# Modeling the economic impact of different vial-opening thresholds for measles-containing vaccines

**DOI:** 10.1016/j.vaccine.2019.03.017

**Published:** 2019-04-17

**Authors:** Patrick T. Wedlock, Elizabeth A. Mitgang, Assaf P. Oron, Brittany L. Hagedorn, Jim Leonard, Shawn T. Brown, Jennifer Bakal, Sheryl S. Siegmund, Bruce Y. Lee

**Affiliations:** a HERMES Logistics Modeling Team, Baltimore, MD & Pittsburgh, PA, United States; b Global Obesity Prevention Center (GOPC) at Johns Hopkins University, Johns Hopkins Bloomberg School of Public Health, Baltimore, MD, United States; c Institute for Disease Modeling, Bellevue, WA, United States; d Pittsburgh Supercomputing Center, Carnegie Mellon University, Pittsburgh, PA, United States; e McGill Centre for Integrative Neuroscience, McGill Neurological Institute, McGill University, Montreal, Canada

**Keywords:** Measles vaccine, Vaccine supply chain, Presentation, Policy

## Abstract

**Introduction:**

The lack of specific policies on how many children must be present at a vaccinating location before a healthcare worker can open a measles-containing vaccine (MCV) – i.e. the *vial-opening threshold* – has led to inconsistent practices, which can have wide-ranging systems effects.

**Methods:**

Using HERMES-generated simulation models of the routine immunization supply chains of Benin, Mozambique and Niger, we evaluated the impact of different vial-opening thresholds (none, 30% of doses must be used, 60%) and MCV presentations (10-dose, 5-dose) on each supply chain. We linked these outputs to a clinical- and economic-outcomes model which translated the change in vaccine availability to associated infections, medical costs, and DALYs. We calculated the economic impact of each policy from the health system perspective.

**Results:**

The vial-opening threshold that maximizes vaccine availability while minimizing costs varies between individual countries. In Benin (median session size = 5), implementing a 30% vial-opening threshold and tailoring distribution of 10-dose and 5-dose MCVs to clinics based on session size is the most cost-effective policy, preventing 671 DALYs ($471/DALY averted) compared to baseline (no threshold, 10-dose MCVs). In Niger (median MCV session size = 9), setting a 60% vial-opening threshold and tailoring MCV presentations is the most cost-effective policy, preventing 2897 DALYs ($16.05/ DALY averted). In Mozambique (median session size = 3), setting a 30% vial-opening threshold using 10-dose MCVs is the only beneficial policy compared to baseline, preventing 3081 DALYs ($85.98/DALY averted). Across all three countries, however, a 30% vial-opening threshold using 10-dose MCVs everywhere is the only MCV threshold that consistently benefits each system compared to baseline.

**Conclusion:**

While the ideal vial-opening threshold policy for MCV varies by supply chain, implementing a 30% vial-opening threshold for 10-dose MCVs benefits each system by improving overall vaccine availability and reducing associated medical costs and DALYs compared to no threshold.

## 1 Introduction

The lack of specific policies on how many children must be present at a vaccinating location before a healthcare worker can open a measles-containing vaccine (MCV) – referred to as the *vial-opening threshold* – has led to variable and inconsistent practices within and between countries [1], [2]. Due to the complexity of vaccine supply chains [3], vial-opening threshold decisions for MCV, which must be discarded within six hours of opening, may have reverberating effects on the entire system.

While various MCV vial-opening threshold policies have been advocated [1], [2], [4], [5], the direct effects of these policies are not well documented and their effects on the entire vaccine supply chain system are unknown. Advocates for no vial-opening threshold (i.e. opening an MCV vial for any number of children) want to reduce missed opportunities to vaccinate at the expense of higher MCV open-vial wastage [2], [5]. Conversely, advocates for strict vial-opening thresholds (requiring six or more children to be present before a 10-dose MCV vial is opened) want to preserve MCV vials for larger vaccination sessions and reduce the costs associated with wastage and procurement of additional vaccines [1], [4]. Such existing policies for strict vial-opening measures are often a response to specific wastage targets established for immunization programs of Gavi-eligible countries [1], [2]. While few studies have pointed to the effects of these policies on MCV availability, wastage, and costs for specific supply chains, there is no evidence for how these or any other vial-opening threshold policies may affect the entire supply chain system – particularly the availability of other routine immunizations – or apply across multiple heterogenous supply chains.

As vaccine supply chains are complex, a systems approach is needed to evaluate how different vial-opening thresholds and MCV presentations can affect not just MCV, but the entire vaccine supply chain system and, in turn, the health of the population [6], [7], [8], [9], [10], [11], [12]. In this paper, we use the Highly Extensible Resource for Modeling Supply Chains (HERMES) supply chain modeling software to simulate the operational, economic, and clinical effects of different MCV vial-opening threshold policies (no threshold, 30% of doses must be used, and 60% of doses must be used) and MCV presentations (10-dose, 5-dose) across vaccine supply chains in the countries of Mozambique, Benin, and Niger.

## 2 Methods

### 2.1 HERMES supply chain modeling software

HERMES is a discrete event simulation modeling software platform that can generate computer simulation models of supply chains. A HERMES-generated supply chain model is a virtual representation of all storage facilities (including refrigerators and freezers), transport routes (including shipping policies, vehicles, drivers, and per diems), healthcare personnel, vaccines, administration points (including vial opening policies and population demand), and includes the associated costs for each component. Previous publications have described HERMES models in detail [13], [14].

### 2.2 HERMES vaccine supply chain models: Benin, Mozambique and Niger

Our team used HERMES simulation models of the routine immunization supply chains of Benin [15], [16], [17], Mozambique [18], and Niger [19], [20]. The routine immunization programs in these countries are eligible for Gavi support [21] and their supply chains provide a range of different characteristics found in Gavi-supported vaccine supply chains [22]. The supply chain model of Benin is comprised of one national store, seven department stores, 80 communes, and 763 health posts and was developed using cold chain and population data from 2012. The supply chain model of Mozambique is comprised of one national store, ten provincial stores, 104 district stores, 1428 health facilities, and 254 mobile brigades and was developed using cold chain and population data from 2014. The supply chain model of Niger includes one central store, eight regional stores, 42 district stores, and 695 integrated health centers and was developed using cold chain data from 2011 and population data from 2015.

Benin’s routine immunization program provides one dose of measles (M) vaccine, while Mozambique and Niger’s routine programs provide two doses of measles-rubella (MR) vaccine. Each country has set a 40% maximum wastage rate for MCVs [23], [24], [25]. The vaccines modeled reflect the current EPI national immunization schedule for each country as described in Appendix A.

In HERMES, the expected demand per session – referred to as average session size – is determined by dividing the annual demand at a health facility (i.e. the total number of people seeking vaccination at a certain clinic for a certain vaccine over the course of one year) by session frequency (i.e. the number of vaccination sessions held annually), both of which are model inputs (see equation below). Data for these inputs was collected during construction of the model with direct input from country partners.

$$N_{\mathrm{per}\;session}^{P}=N_{\mathrm{per}\;year}^{P}/N_{\mathrm{per}\;year}^{\mathrm{session}}$$

To better simulate reality, HERMES employs a stochastic model of consumer arrival. For each session, HERMES calculates the actual session size by randomly drawing from a Poisson distribution, whose mean parameter is the average session size.

### 2.3 Measles-containing vaccine (MCV) vial-opening threshold policies and presentations

In HERMES, a vaccine vial is opened for any number of people unless the user sets a policy dictating the number of people that need to be present in a vaccination session before a vaccine is opened for use. In each country, we modeled three vial-opening threshold policies to capture a range of existing policies and practices across different immunization programs.

• *Instituting no vial-opening threshold policy* allowed MCVs to be opened for one or more children. This practice has high potential for MCV wastage, but is advocated for in literature and guidelines as a potential solution to improving MCV coverage and reducing missed opportunities to vaccinate [2], [5].
• *Instituting a 30% vial-opening threshold policy* meant that MCVs would be opened only if the demand in a given session would use 30% of MCV doses, i.e. a 10-dose MCV would be opened if at least three children were present and a 5-dose MCV would be opened if at least two children were present. This threshold applies to each MCV vial needed such that if a vaccination session has 11 or 12 children, only one 10-dose vial of MCV would be opened. This policy is not referenced as current practice in any countries but serves as an intermediary scenario for the purpose of this study.
• *Instituting a 60% vial-opening threshold policy* restricted the use of MCVs further, i.e. 10-dose MCV vials would be opened if at least six children were present and a 5-dose MCV would be opened if at least 3 children were present. This more restrictive practice is commonly identified as a way to meet MCV wastage targets, and is practiced by healthcare workers in a number of countries [1], [4].

In addition to simulating three different vial-opening thresholds, we considered the effects of these policies across different presentations of MCV. Currently, 10-dose MCV vials are used, although UNICEF is supplying 5-dose MCV vials as of 2018 [26]. As countries consider introducing 5-dose MCV, which has a higher volume- and price-per-dose, into their supply chain systems, we consider the effects of different vial-opening thresholds across three different MCV presentation scenarios:

• Distributing 10-dose MCV vials to all vaccinating locations in a country (current practice)
• Distributing 5-dose MCV vials to all vaccinating locations in a country
• Distributing a mix of 10-dose and 5-dose MCV vials according to the average session size (see Section 2.2) of each vaccinating location, i.e. locations with average session sizes of 5 or fewer children receive 5-dose MCV, while locations with average session sizes of 6 or more children receive 10-dose MCV

Lastly, in addition to Benin, Niger, and Mozambique’s vaccine supply chains, we ran each of the above scenarios using an unconstrained Niger supply chain model. To do this, we ran a gap analysis in HERMES that calculated all of the necessary storage and transport space needed to achieve 100% vaccine availability and added this equipment to the Niger model. We then tested the effects of different MCV vial-opening thresholds and presentations on this hypothetical, unconstrained system.

### 2.4 Clinical and economic model for EPI vaccine-preventable diseases

In addition to calculating the effects of different MCV vial-opening threshold policies and presentations on supply chain operations and costs, we developed an economic and clinical outcomes model to calculate the incremental cost-effective ratio (ICER) of each scenario. This economic model takes a health systems perspective, which includes health care costs paid by third-party payers and out-of-pocket by patients [27]. We estimated the cost of healthcare delivery, assuming that patients would pay at-cost without significant profit margin. To this end, the model includes the cost of supply chain logistics for vaccines, vaccine procurement prices, and direct medical treatment costs associated with the vaccine-preventable infections of each modeled disease. We utilized disease incidence data from the Institute for Health Metric’s Global Burden of Disease (GBD) and the most recent authoritative meta-analysis from West Africa (see Appendix B for incidence data for each disease and relevant citations) to calculate vaccine-preventable infections (see Section 2.5 below).

We calculated total direct medical costs using a bottom-up approach, including the cost of visits to outpatient health posts, the daily cost of hospitalization, and pharmaceutical costs. We did not include the cost of diagnostics, complex procedures (that are often not available in the modeled countries), medical care to manage chronic disability, nor the indirect patient cost of time spent seeking care.

To avoid underestimating the cost of disease burden, we assumed that all individuals with acute symptoms would seek and receive the correct care for their condition, as defined by Medecins Sans Frontieres (MSF) guidelines for working in low-resource settings [28].

We calculated hospitalization costs by multiplying the probability of admission with the average length of stay [29] and the country-specific cost per day for inpatient care. Health post visit costs were calculated based on one visit per infection for those individuals not hospitalized. Unit costs per day and per visit were country-specific and based on the WHO’s CHOICE estimates for public facilities, updated to 2017 GDP and rural/urban population mix [30].

For all diseases except tuberculosis (TB), we based pharmaceutical costs on UNICEF’s published international indicative prices, assuming that public health providers would purchase supplies in reasonable bulk sizes when it affords lower prices. The price of immunoglobulin for tetanus treatment was taken from the WHO’s reported average cost [31]. We applied costs for symptom control drugs (i.e. fever reduction, pain management) to every infection and treatment drugs (i.e. antibiotics) to every hospitalization. We calculated dosing levels based on the amount required to care for a 20 kg 5-year old, since the largest portion of disease burden for most vaccine-preventable diseases is in young children.

For TB, we utilized pharmaceutical costs from the most recent cost catalog by the Stop TB program [32], a partner of the Global Fund to fight AIDS, Tuberculosis, and Malaria and is the largest funder of TB treatment globally. Dosages were calculated based on a 60 kg adult, since most TB burden is latent until later in life. We based our calculations on the WHO’s recommended regimen for new patients of daily administration for 2 months with HRZE and 3-times weekly administration for 4 months of HR in fixed dose combination formulations (FDC) [33].

### 2.5 Simulation scenarios for MCV vial-opening thresholds and presentations

For each country, we simulated nine scenarios pairing one of three MCV vial-opening thresholds with one of three MCV vial presentation scenarios (as described in Section 2.3). In the baseline scenario, 10-dose MCV vials were opened for any number of children (i.e. no vial-opening threshold). In each scenario, running the HERMES model determined how many vaccine-preventable infections occurred in the population:

$$\mathrm{Vaccine}-preventable\;infections\;\left(VPI\right)=\left(Missed\;Vaccinations\times {\mathrm{Incidence}}_{\mathrm{UnvaccinatedPopulation}}\right)+\left(Total\;Vaccinated\times {\mathrm{Incidence}}_{\mathrm{VaccinatedPopulation}}\right)$$

The following formulas then determined the medical costs and disability-adjusted life years (DALYs) from the infections prevented:

Directmedicalcosts=VPI×Averagemedicalcostperinfection

DALYs=VPI×AverageDALYsperinfection

We assessed the impact of uncertainty in the model parameters by using a Monte Carlo simulation to simultaneously vary the parameters presented in Appendix B. The simulation sampled values for the DALYs per case, direct medical cost per case, vaccine effectiveness, and disease incidence. Ranges for DALYs, vaccine effectiveness, and incidence were simulated as triangular distributions across their 95% confidence interval (CI) values. Uncertainty ranges for hospitalization day rates and outpatient visit unit costs were estimated based on previous CIs calculated for these costs in Mozambique [30]. Pharmaceutical prices are established on the international market, so we did not simulate uncertainty in their pricing. We generated 1000 stochastic results for each disease-country combination and then aggregated the results and calculated the simulated 95th percentile prediction intervals.

Additionally, we calculated the incremental cost-effectiveness ratio (ICER) of each experimental scenario compared to the scenario with the next-most DALYs (i.e. next best scenario). Scenarios were considered dominated if there was an alternative scenario which resulted in both lower costs and fewer DALYs.

ICER=((Logistics+procurement+medicalcostsincurredinexperimentalscenario)-(logistics+procurement+medicalcostsincurredinnextbestscenario))/(DALYsincurredinnextbestscenario-DALYsincurredinexperimentalscenario)

Cost-effectiveness is defined as an ICER ratio <3× gross domestic product (GDP) per capita, while highly cost-effective is <1× GDP per capita. In the last reported year (2017) GDP per capita was $829.8 in Benin, $378.1 in Niger, and $415.7 in Mozambique.

## 3 Results

Each simulation is run at a daily resolution over one year; results reflect the average of 23 runs. All costs are reported in 2018 USD.

### 3.1 Requiring HCWs to open MCVs for any number of children per session (No vial-opening threshold)

**Fig. 1:**
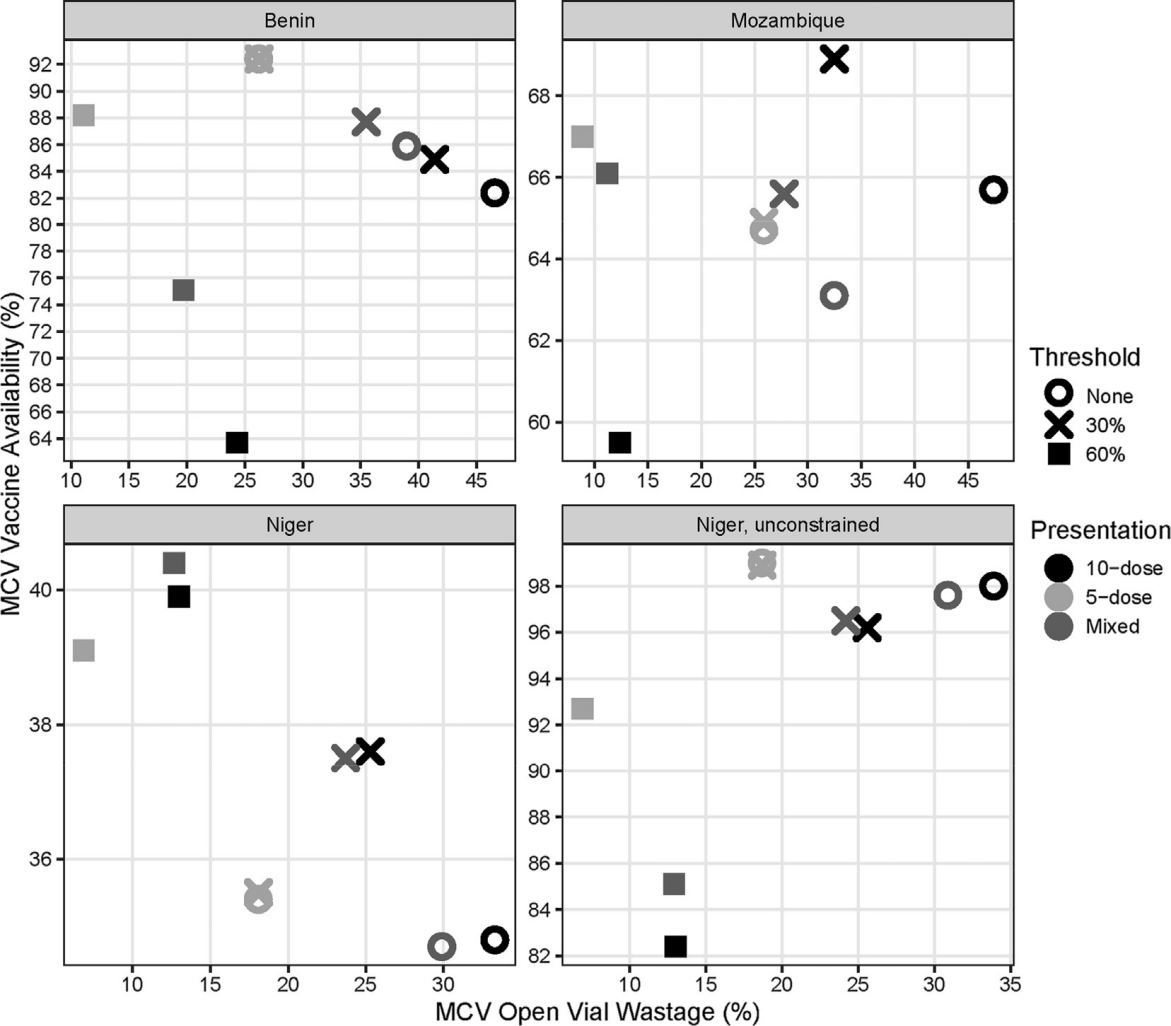
Measles-containing vaccine (MCV) availability vs. MCV open vial wastage.

Across all three vaccine supply chains, instituting no vial-opening threshold for 10-dose MCV (baseline) resulted in the highest MCV wastage (33–47%) compared to all other scenarios. As shown in Fig. 1, opening 10-dose MCV vials for any number of children results in decreased MCV vaccine availability (i.e. successful immunizations administered to patients as a percentage of total immunizations needed) compared to other scenarios, except in Niger when all of the storage and transport constraints are removed. Given the existing cold chain and transport constraints in each system (Table 1), many vaccinating locations will not be able to maintain enough stock between shipments if an MCV vial is opened for even one or two children. Further, since each vaccinating location in these countries has an average session size of three or more children, using MCV for one or two children means that a stockout would likely occur during a larger session.

**Table 1:** Baseline characteristics of benin, mozambique, and niger vaccine supply chains.

Indicator	Benin	Mozambique	Niger
MCV Session Size (Median, IQR)	5 (5–6)	3 (2–7)	9 (6–10)
Number of vaccinating locations with average MCV session sizes^a^ of:			
1–2 children	0	649	22
3–5 children	452	464	90
6–10 children	204	245	334
11–12 children	2	88	90
13–15 children	0	52	75
16–20 children	0	46	33
20+ children	0	93	0

Peak storage capacity utilization* (averaged across all storage devices in a given level)			
Top level	93%	99%	99%
Second level	38%	61%	77%
Third level	39%	65%	76%
Fourth level	8%	28%	28%
Peak transport capacity** utilization (averaged across all routes)	98%	191%	362%

a Average MCV session size refers to the average number of children per session over the course of a year. Clinics may, however, have more or fewer children on any given day. Please refer to the Methods for further details.

* Peak storage capacity utilization is the maximum percentage of available storage capacity occupied by products at any time.

** Peak transport capacity utilization is the maximum percentage of available transport capacity needed to complete any shipment.

**Fig. 2:**
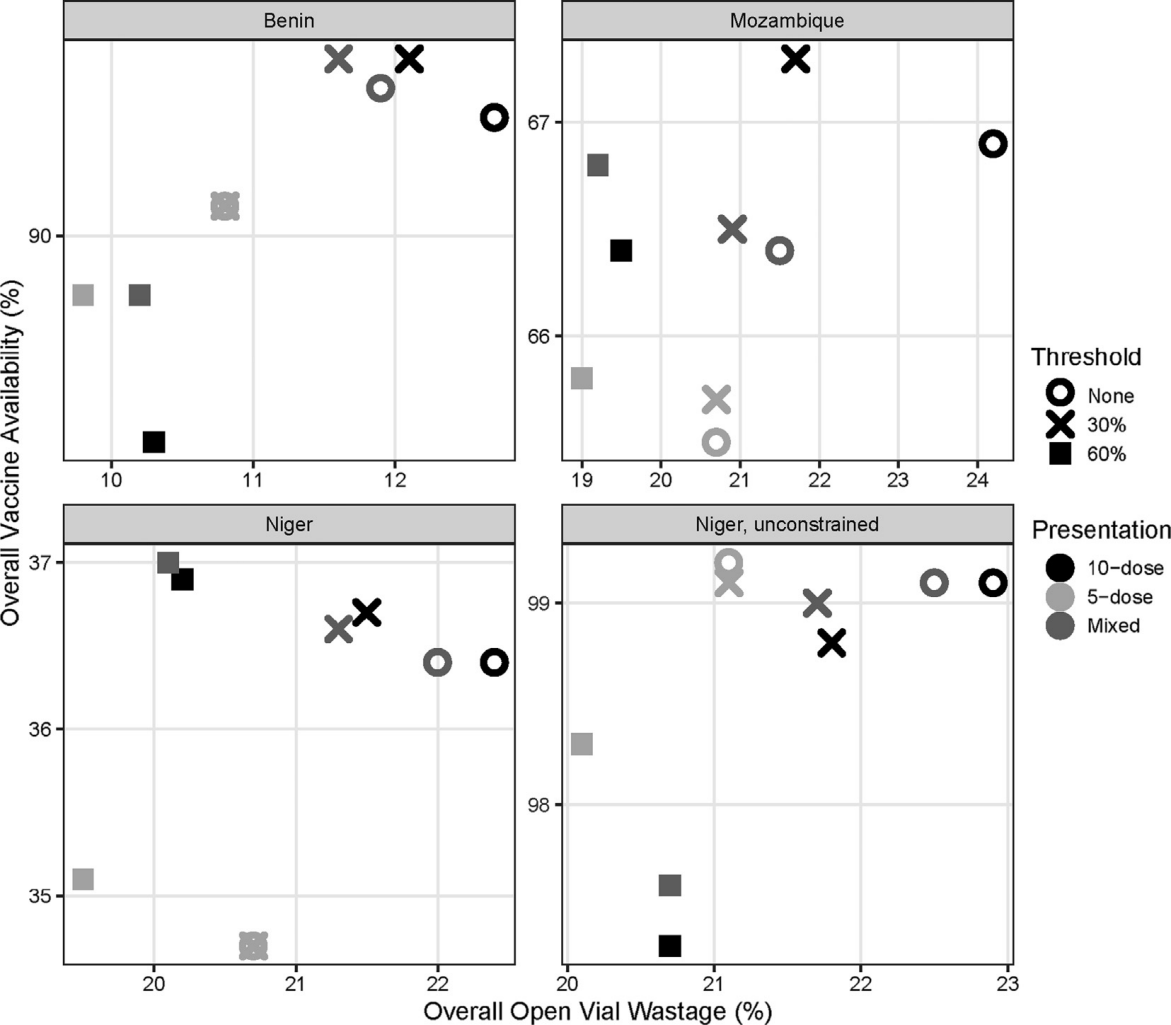
Overall vaccine availability vs. total open vial wastage.

**Fig. 3:**
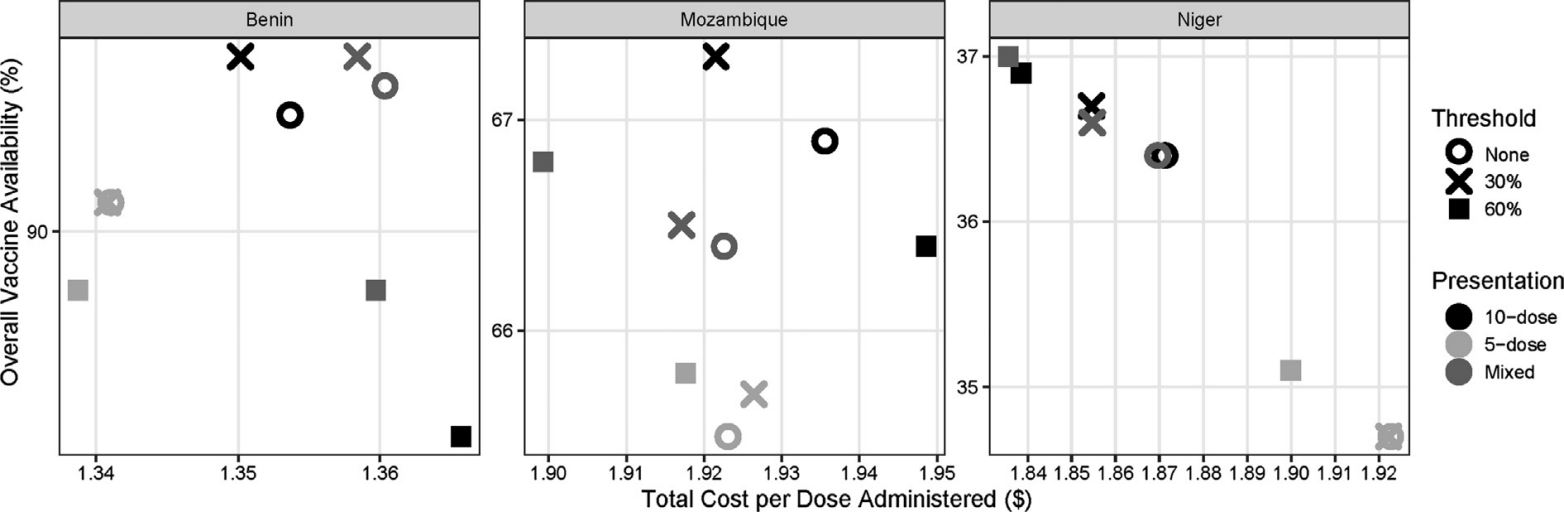
Overall vaccine availability vs. total cost per dose administered.

**Fig. 4:**
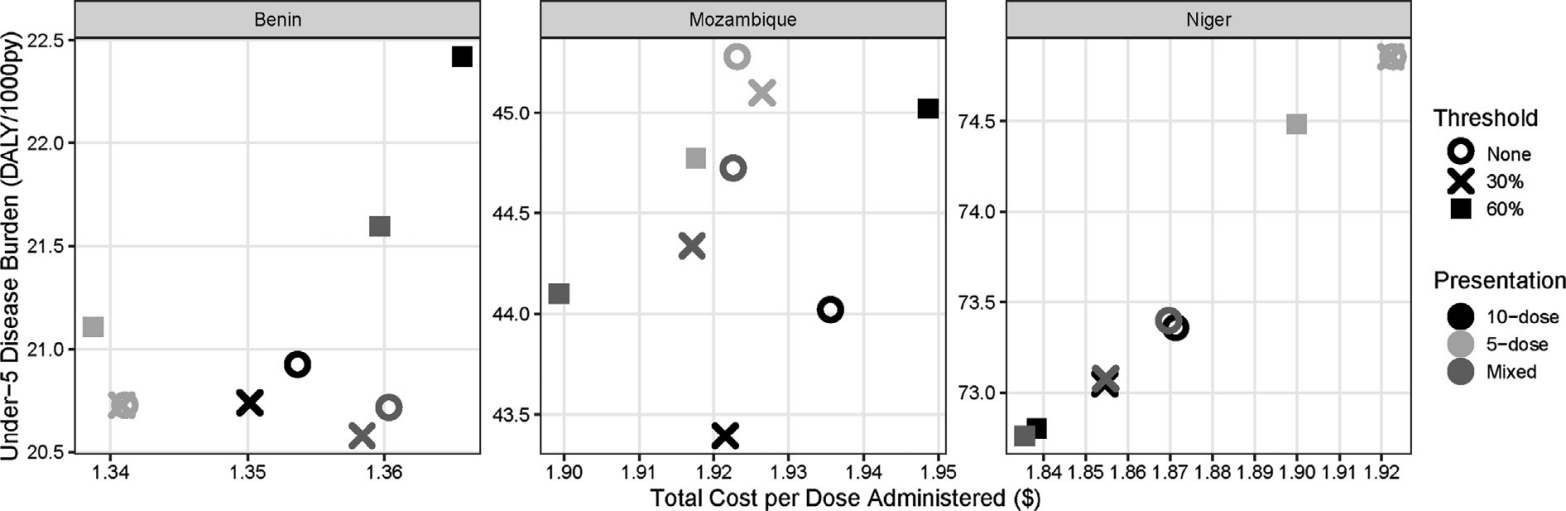
Overall burden of vaccine-targeted diseases vs. total cost per dose administered.

In terms of overall vaccine availability (Figs. 2 and 3), however, this policy outperforms many others, particularly in Mozambique and Niger, which both have highly constrained cold chain systems. When 10-dose vials are used everywhere, the total volume of MCV flowing through the system is smaller than when 5-dose MCV or a mix of 5-dose and 10-dose MCV are introduced. As such, this policy does not impede the flow of other vaccines. In Mozambique, in particular, this policy results in the second-fewest DALYs from all EPI vaccine-preventable diseases out of any scenario (see Fig. 4).

Conversely, switching to 5-dose vials everywhere and maintaining no vial-opening threshold only benefits Benin, which has the fewest cold chain constraints among these systems. Implementing no vial-opening threshold using 5-dose MCV vials reduces both total costs (-$146,094) and DALYs ( −380) in Benin compared to using 10-dose MCV vials without a threshold. Because a majority of locations in Benin (68%) have fewer than five children on average, matching the number of MCV doses-per-vial to session size substantially reduces MCV wastage (−21%). By decreasing the amount of extra MCV otherwise needed to account for wastage, the cold chain storage capacity can handle the increase in MCV volume associated with 5-dose vials. In both Mozambique and Niger, switching to 5-dose vials without a threshold exacerbates existing cold chain storage constraints, resulting in an increase in DALYs of 6178 and 7247, respectively.

Tailoring vaccine vials by session size and maintaining no vial-opening threshold improved total vaccine availability in Benin compared to using 10-dose vials (1.6%) and resulted in fewer DALYs than using 10-dose or 5-dose vials everywhere. In both Mozambique and Niger, tailoring MCV presentations reduced DALYs compared to using 5-dose vials everywhere, but did not improve the system compared to using 10-dose vials everywhere. In both of these supply chains, extensive cold chain constraints were increased when any number of 5-dose vials were introduced without a vial-opening threshold.

By completely removing cold chain constraints, as shown in the hypothetical unconstrained Niger scenario, maintaining no vial-opening threshold results in the highest vaccine availability out of all scenarios (Fig. 1, Fig. 2). In this system, even with high vaccine wastage, there is enough space available in refrigerators and vehicles to maintain stock levels and prevent stockouts from occurring. As such, opening a vial for even one child will result in vaccine availability of 99%. Any vial-opening threshold will increase missed opportunities and reduce vaccine availability.

### 3.2 Requiring HCWs to wait for 3+ children to open 10-dose MCV and 2+ children to open 5-dose MCV (30% vial-opening threshold)

Introducing a 30% vial-opening threshold policy for 10-dose vials everywhere reduces MCV open vial wastage by 6–14%. A 30% vial-opening threshold policy means that a healthcare worker must wait until three or more children are present before opening a 10-dose MCV vial or two or more children are present for a 5-dose MCV vial. As indicated in Table 1, only Mozambique has a large proportion of vaccinating locations with average MCV session sizes of one to two children (39%), meaning that both Benin and Niger will be able to reduce high MCV open vial wastage without substantially increasing missed opportunities to vaccinate. Even in Mozambique, however, preserving 10-dose vials for three or more children reduces stockouts of MCV for larger sessions and leads to an improvement in both MCV availability (+3%) and total vaccine availability (+0.4%). As shown in Fig. 2 and Table 3, this threshold policy results in the highest total vaccine availability and fewest DALYs in Mozambique.

In Benin and Niger, implementing a 30% vial-opening threshold with 10-dose MCV vials reduces MCV wastage and improves MCV availability (+3%) and total vaccine availability (0.1–0.3%). As both of these countries have few vaccinating locations with less than three children on average per MCV session (Table 1), switching to a 30% threshold reduces MCV wastage while preserving a finite number of MCV vials for larger sessions. Further, as fewer MCV vials are wasted, fewer MCV vials are procured, freeing up constrained transport space for other vaccines (see Table 2).

**Table 2:** Doses administered, vaccine availability, and open vial wastage by country.

	Doses administered		Vaccine availability		Open vial wastage	
Scenarios by supply chain	Total	MCV	Total	MCV	Total	MCV
*10-dose MCV; no OVT*						
Benin	5,334,809	265,998	90%	82%	13%	47%
Mozambique	13,441,544	1,410,318	67%	66%	24%	47%
Niger	6,444,036	656,225	36%	35%	22%	33%
Niger unconstrained	17,595,855	1,850,138	99%	98%	23%	34%

*10-dose MCV; 30% OVT*						
Benin	5,342,655	274,068	91%	85%	12%	41%
Mozambique	13,515,147	1,479,925	67%	69%	22%	33%
Niger	6,496,168	709,332	37%	38%	22%	25%
Niger unconstrained	17,541,208	1,817,778	99%	96%	22%	26%

*10-dose MCV; 60% OVT*						
Benin	5,267,596	205,680	89%	64%	10%	24%
Mozambique	13,331,819	1,276,776	66%	60%	20%	12%
Niger	6,543,966	752,043	37%	40%	20%	13%
Niger unconstrained	17,264,271	1,557,254	97%	82%	21%	13%

*5-dose MCV; no OVT*						
Benin	5,317,185	298,301	90%	92%	11%	26%
Mozambique	13,149,757	1,388,772	66%	65%	21%	26%
Niger	6,145,535	668,292	35%	35%	21%	18%
Niger unconstrained	17,603,820	1,871,815	99%	99%	21%	19%

*5-dose MCV; 30% OVT*						
Benin	5,318,770	298,048	90%	92%	11%	26%
Mozambique	13,191,958	1,393,899	66%	65%	21%	26%
Niger	6,146,658	668,567	35%	36%	21%	18%
Niger unconstrained	17,572,176	1,869,061	99%	99%	21%	19%

*5-dose MCV; 60% OVT*						
Benin	5,296,215	284,585	90%	88%	10%	11%
Mozambique	13,215,400	1,439,548	66%	67%	19%	9%
Niger	6,212,193	737,245	35%	39%	20%	7%
Niger unconstrained	17,446,535	1,753,470	98%	93%	20%	7%

*Mixed MCV dose; no OVT*						
Benin	5,338,233	277,566	91%	86%	12%	39%
Mozambique	13,328,273	1,355,098	66%	63%	22%	33%
Niger	6,436,502	653,357	36%	35%	22%	30%
Niger unconstrained	17,580,582	1,846,439	99%	98%	23%	31%

*Mixed MCV dose; 30% OVT*						
Benin	5,342,580	283,052	91%	88%	12%	36%
Mozambique	13,362,768	1,407,654	67%	66%	21%	28%
Niger	6,489,468	706,422	37%	38%	21%	24%
Niger unconstrained	17,570,946	1,825,521	99%	97%	22%	24%

*Mixed MCV; 60% OVT*						
Benin	5,297,278	242,238	90%	75%	10%	20%
Mozambique	13,409,183	1,418,543	67%	66%	19%	11%
Niger	6,549,055	762,785	37%	40%	20%	13%
Niger unconstrained	17,336,398	1,610,272	98%	85%	21%	13%

A moderate (30%) vial-opening threshold policy for 5-dose vials everywhere results in similar effects on the vaccine supply chain as using 5-dose vials with no vial opening threshold policy. Despite the decrease in MCV open vial wastage of 7–16% when switching from 10-dose vials to 5-dose vials under this policy, the larger vaccine volume in the system reduces MCV availability by 3–4% in the more constrained supply chains, Mozambique and Niger. In Benin, however, where constraints are fewest and no vaccinating locations have average session sizes of one or two children, using 5-dose MCV vials with a 30% threshold reduces both total costs (-$145,264) and DALYs ( −381) compared to the baseline policy of no threshold using 10-dose vials. While this isn’t the most cost-effective policy for Benin, it does improve the system.

The most cost-effective policy in Benin involves implementing a 30% vial-opening threshold using a mix of 10-dose and 5-dose MCV vials. In this scenario, 5-dose MCV is distributed to all vaccinating locations with fewer than five children per MCV session on average, and 10-dose MCV to all other locations. As shown in Table 1, the distribution of children per session is clearly divided between three to five children (69%) and six to ten children (31%). As such, tailoring vaccine vials with a 30% threshold prevents 671 DALYs compared to baseline at a cost/DALY averted of $471.10 (Table 3).

**Table 3:** Incremental cost-effectiveness ratio (ICER) of each combination of MCV vial-opening threshold and presentation for benin, mozambique, and niger.

**Country**	**Scenario Number**	MCV*Vial-Opening Threshold	**MCV Presentation**	**DALYs**	Total Costs**	**$ per DALY Averted**
**Benin**	1	0%	10-dose	40,478	$ 8,620,704	dominated by scenarios 2, 4-6
	2	0%	5-dose	40,097	$ 8,474,610	minimum cost
	3	0%	Mixed	40,076	$ 8,633,256	dominated by scenario 6
	4	30%	10-dose	40,120	$ 8,590,508	dominated by scenarios 2, 5
	5	30%	5-dose	40,097	$ 8,475,440	$ 5,750.87
	6	30%	Mixed	39,807	$ 8,612,227	$ 471.10
	7	60%	10-dose	43,366	$ 8,764,517	dominated by all scenarios
	8	60%	5-dose	40,830	$ 8,475,237	dominated by scenario 2
	9	60%	Mixed	41,772	$ 8,674,217	dominated by scenarios 1-6, 8
**Mozambique**	1	0%	10-dose	215,921	$ 31,609,206	dominated by scenario 4
	2	0%	5-dose	222,099	$ 31,035,328	dominated by scenario 8
	3	0%	Mixed	219,370	$ 31,406,402	dominated by scenarios 4, 6, 9
	4	30%	10-dose	212,840	$ 31,342,759	$ 85.98
	5	30%	5-dose	221,215	$ 31,132,486	dominated by scenarios 8, 9
	6	30%	Mixed	217,476	$ 31,239,636	dominated by scenario 9
	7	60%	10-dose	220,827	$ 31,967,851	dominated by scenarios 1, 3,4, 6, 8,9
	8	60%	5-dose	219,611	$ 30,923,595	minimum cost
	9	60%	Mixed	216,309	$ 31,044,511	$ 36.62
**Niger**	1	0%	10-dose	354,439	$ 15,775,616	dominated by 4, 6,7, 9
	2	0%	5-dose	361,686	$ 15,602,676	dominated by scenarios 5, 8
	3	0%	Mixed	354,627	$ 15,752,997	dominated by scenarios 4, 6,7, 9
	4	30%	10-dose	352,936	$ 15,743,208	dominated by scenarios 7, 9
	5	30%	5-dose	361,682	$ 15,601,407	dominated by scenario 8
	6	30%	Mixed	353,078	$ 15,733,775	dominated by scenarios 7, 9
	7	60%	10-dose	351,734	$ 15,709,481	dominated by scenario 9
	8	60%	5-dose	359,865	$ 15,562,710	minimum cost
	9	60%	Mixed	351,542	$ 15,696,334	$ 16.05

* MCV = Measles-containing vaccine

** Total costs include all logistics costs, vaccine procurement costs, and medical costs

In Niger, tailoring vaccines and using a 30% policy improves both total and MCV availability compared to baseline, but does not match the benefits of simply using 10-dose MCV vials everywhere with a 30% vial-opening policy. And in Mozambique, introducing a large number of 5-dose vials into the system with existing constraints results in an increase in cold chain constraints and a decrease in both total and MCV availability compared to baseline.

### 3.3 Requiring HCWs to wait for 6+ children to open 10-dose MCV and 3+ children to open 5-dose MCV (60% vial-opening threshold)

Introducing a 60% vial-opening threshold policy across each supply chain system results in the largest decrease in open vial wastage compared to the other threshold policies, but overly restricts access to MCV for a number of vaccinating locations, particularly in Benin and Mozambique where a majority of all sessions have fewer than six children on average. In Niger, however, where the median session size is nine and a majority of locations have more than six children on average, setting this restrictive policy improves MCV availability across all MCV presentation scenarios, while improving total vaccine availability when either 10-dose MCV vials or a mix of 10- and 5-dose MCV vials are used.

In Benin, 69% of locations have fewer than six children per MCV session on average. While a 30% vial-opening threshold balances the tradeoffs between reducing wastage and preserving vaccines for larger sessions, the 60% threshold overly restricts MCV usage, particularly when 10-dose MCV vials are used everywhere. While MCV wastage decreases by 23%, MCV availability decreases by 18%, and the number of DALYs for all EPI vaccine-preventable diseases increases by 2888. When 5-dose vials are used with this policy, MCV availability improves (though not as much as 0% or 30% thresholds), but total vaccine availability decreases due to the increase in cold chain constraints. When a mix of MCV presentations are used, allowing 5-dose vials to be opened for three or more children and 10-dose vials to be opened for six or more children, both MCV availability and total availability increase compared to baseline. Yet, this policy does not outperform the 0% and 30% thresholds when using a mix of presentations.

In Mozambique, the 60% threshold is too restrictive when using 10-dose MCV everywhere, resulting in a 5% decrease in total vaccine availability and an increase of 4906 DALYS compared to no vial opening threshold. Additionally, as occurred with the other threshold policies when using 5-dose vials or a mix of presentations in Mozambique, the influx of 5-dose vials overly increased the strain on the system. While the 60% threshold improved MCV availability for 5-dose vials and a mix of vials compared to baseline, total vaccine availability decreased with the additional constraints, resulting in a net-negative outcome for the supply chain system.

In Niger, which has the largest median session size of the three modeled supply chains (nine children), the most cost-effective policy is instituting a 60% vial-opening threshold and tailoring MCV presentations. This policy results in a decrease in total costs of $79,281 and DALYs of 2897 at a cost/DALY averted of $16.05. This policy improves the availability of all vaccines by closely matching the doses per vial to session size and preserving vials for larger sessions. While not the most cost-effective overall, instituting a 60% vial-opening threshold while using 10-dose MCV vials everywhere produces nearly the same decrease in both DALYs (−2705) and total costs (-$66,134) compared to tailoring presentations and using a 60% policy. This policy does not have the additional advantage of distributing 5-dose MCV vials to those locations with five or fewer children on average but does reduce MCV wastage and preserve MCV vials for larger sessions. This not only improves MCV availability but frees up constrained cold chain space for other vaccines, resulting in a benefit to total vaccine availability.

## 4 Discussion

Our results show that implementing any vial-opening threshold policy for MCV can have numerous system-wide effects, while the vial-opening threshold policy that maximizes vaccine availability and cost-savings vary between countries. By considering certain aspects of each system – including existing cold chain constraints, average demand per session, and the health outcomes of each vaccine-preventable infection – policymakers can make an informed decision on the appropriate MCV threshold policy and presentation for their system.

Cold chain constraints play a major role in mediating the effects of different vial-opening thresholds and vial presentation combinations for the vaccine supply chain system. When no cold chain constraints exist, as in the unconstrained Niger scenario, the most beneficial policy in terms of increasing vaccine availability is to open a multidose MCV for any number of children. Setting any threshold policy (e.g. 30% or 60%) in an unconstrained system reduces availability to below 100%. However, many vaccine supply chains are constrained and, even in the least-constrained system (i.e. Benin), setting a 30% vial-opening threshold and preserving MCV vaccines for larger sessions can reduce stockouts and increase vaccine availability. Further, these constraints need to be considered when deciding whether to use 5-dose MCV vials for either part or all of the vaccine supply chain, as the increase in volume-per-dose can offset the decrease in open vial wastage, particularly in highly constrained settings.

In addition to cold chain constraints, the number of children per MCV session has a big impact on the effects of different vial-opening thresholds. Across the three vaccine supply chains modeled, the median number of children per session varied from three to nine. In Benin and Mozambique, which had median session sizes of more than two but fewer than six children, the 30% vial-opening thresholds for 10-dose MCV or a mix of MCV vial sizes were the most beneficial, while switching to a 60% threshold was far too restrictive. In Niger, however, where the median session size for MCV was nine children, the 60% threshold performed well, preserving a finite number of MCV vials for larger sessions.

Beyond the effects of these vial-opening thresholds and varying presentations on MCV availability, these policies need to be considered within the scope of the larger system. In Benin, switching to 5-dose MCV vials and using either no threshold or a 30% threshold led to an improvement in MCV availability of 10% compared to using 10-dose MCV with no threshold. This was a result of decreasing MCV wastage by nearly 21%. However, this policy and presentation combination also introduced new constraints into the cold chain system and slightly reduced the availability of all other vaccines. While this policy still led to an overall improvement in vaccine availability and DALYs compared to the baseline policy, the most cost-effective policy included tailoring vaccine vial sizes in order to reduce the additional cold chain constraints that occur with 5-dose MCV vials.

The interplay between vial-opening policies and MCV presentation is discussed in WHO’s Measles and Rubella Initiative 2013 guidance document [34]. This guidance acknowledges the potential storage space constraint with 5-dose vials and concludes that in storage-constrained settings opening a 10-dose vial for any number of children is the simplest option. This reflects a broad consensus among global health bodies and researchers, advocating for vaccinate-all-comers policies and discouraging more restrictive local policies.

However, opening a vaccine vial for every child often conflicts with the programmatic goals of achieving low wastage rates. As demonstrated in our simulations, a very restrictive policy (60%) may be at odds with the expected demand per session across a number of vaccinating locations. For healthcare workers vaccinating in these locations, the choice between achieving target wastage rates or reducing missed opportunities is difficult. Our simulations show that these policies are mechanistically influenced by average session size at each location and, left unconsidered, setting low target wastage rates and restricting MCV access can have negative systems effects.

Balancing these tradeoffs is critical to improving the function of an immunization system. Countries with access to data on average session sizes and cold chain constraints may be able to refine MCV vial-opening threshold policies and presentations to better match their system. In Benin, where the system is less constrained and average session sizes are neatly aligned with 10- and 5-dose vials, tailoring MCV within the system and instituting a 30% vial-opening threshold can provide substantial additional benefits. In Niger, where the median demand per MCV session is greater than six, setting a more restrictive policy of 60% with 10-dose vials or a mix of 10-dose and 5-dose vials can provide even greater benefit than a 30% vial-opening threshold. Across all supply chain systems, however, a 30% vial-opening threshold provided a balance between reducing MCV wastage and preventing missed opportunities to vaccinate. Instituting this vial-opening threshold as standard practice could improve the function of a number of heterogenous supply chain systems.

## 5 Limitations

While each computational model utilized in this analysis attempts to represent real life, simulations are ultimately simplifications of complex components and processes, and in turn, cannot capture every aspect and outcome. In HERMES, when a person arrives to be vaccinated and is turned away, either due to the vial-opening threshold policy or stockouts, that person does not attempt to be vaccinated again over the course of the simulated year. We did not formally report confidence intervals or uncertainty in our results. The coefficient of variation for simulation endpoints between replicate runs of each scenario was very low at <1%. One could envision incorporating broader sources of uncertainty into the simulation; however, that would require a much larger ensemble of runs, making the simulation computationally prohibitive. We did not consider changing the assignment of a clinic between a 10- or 5-dose vial over the course of the year, nor did we consider providing a mix of MCV presentations to a given clinic.

Secondly, our study applies the same range of vaccine effectiveness (95%-100% efficacy) whether one measles dose is administered (i.e. Benin) or two doses are administered (i.e. Mozambique and Niger). While available literature indicates that the vaccine efficacy of one and two doses falls within this range [35], our assumption has the potential to underestimate the added protection from a second dose.

In addition, the systems modeled in HERMES reflect the supply chains of 2012 in Benin, 2014 in Mozambique, and 2011 in Niger, but we applied the 2018 vaccination list to our simulations. In Niger in particular, in recent years many new vaccines were added to the country’s schedule (3 doses of PCV, 3 doses of rotavirus, and MCV2). The severity of Niger’s constraints is likely overstated since Gavi documents indicate some supply-chain expansion in recent years to accommodate additional vaccines. This is another reason why we chose to simulate a constraint-free version of the Niger system. That said, Niger is still likely the most constrained of the three supply chains, and its simulation results demonstrate what happens in a system or part of any country’s system that is severely constrained.

## 6 Conclusion

While the ideal vial-opening threshold policy for MCV may vary by supply chain, a 30% vial-opening threshold policy for 10-dose MCV vials provides benefits to each system compared to no threshold by reducing MCV open-vial wastage, improving vaccine availability, and reducing associated medical costs and DALYs. Contrary to current guidance, opening a vial for every child often resulted in negative systems effects, while a more restrictive 60% threshold only performed well in Niger, where median session size was high. Policymakers seeking to improve their system should consider the average demand per session and existing cold chain constraints when deciding which vial-opening threshold to implement.

## Conflict of interest

The authors declare that they have no known competing financial interests or personal relationships that could have appeared to influence the work reported in this paper.

## Figures and Tables

**Table:** 

HERMES supply chain model	Vaccine	Doses per vial	Scheduled doses per person	Packed vol (cm^3^) per dose of vaccine	Packed vol (cm^3^) per dose of diluent	Price per vial (2018 USD$)
Benin	BCG	20	1	1.2	0.7	$2.10
	Measles	10	1	2.1	3.1	$3.10
		5		5.48	4.22	$1.75
	OPV	20	4	1	0	$2.40
	PCV13	1	3	12	0	$3.30
	Pentavalent	2	3	11	0	$6.90
	Rotavirus	1	1	17.1	0	$2.25
	IPV	10	1	2.46	0	$8.50
	Tetanus Toxoid	10	2	3	0	$1.29
	Yellow Fever	10	1	2.5	6	$11.50

Mozambique	BCG	20	1	1.2	0.7	$2.10
	Pentavalent	10	3	3	0	$6.90
	Rotavirus	1	2	17.1	0	$2.25
	Measles-Rubella	10	2	2.1	3.1	$6.30
		5		5.48	4.22	$4.10
	OPV	20	4	1	0	$2.40
	PCV10	2	3	4.8	0	$6.10
	Tetanus Toxoid	10	2	3	0	$1.29
	IPV	1	1	14.3	0	$2.80

Niger	BCG	20	1	1.2	0.7	$2.10
	Measles-Rubella	10	2	2.1	0.5	$6.30
		5		5.48	4.22	$4.10
	OPV	20	4	1	0	$2.40
	PCV13	1	3	12	0	$3.30
	Pentavalent	1	3	16.8	0	$1.10
	Rotavirus	1	2	17.1	0	$2.25
	Tetanus Toxoid	10	2	3	0	$1.29
	Yellow Fever	10	1	2.5	6	$11.50

**Table:** 

Disease	Location	Vaccine efficacy (%)	Incidence per 1000 Unvaccinated Population	Incidence per 1000 Vaccinated Population	Direct medical cost per case	DALYs per case	Refs.
Diphtheria	Benin	94 (87–100)	0.009 (0.001–0.031)	0.001 (0–0.002)	$184	39.7 (5.7–75.0)	[28], [36], [37], [38]
	Mozambique		0.009 (0.001–0.029)	0.001 (0–0.002)	$130	36.3 (18.7–70.3)	
	Niger		0.011 (0.001–0.034)	0.001 (0–0.003)	$118	43.6 (6.3–75.0)	

Hepatitis B	Benin	85 (75–95)	165 (64.9–339)	24.8 (5.66–48.7)	$68	2 (1–2)	[28], [37]
	Mozambique		156 (62.5–313)	23.4 (5.21–46.9)	$34	2 (1–2)	
	Niger		138 (57.1–261)	20.7 (4.35–42.9)	$26	2 (1–2)	

Hib	Benin	98 (95–100)	2.55 (0.905–5.56)	0.051 (0–0.226)	$72	10.9 (3.9–30.5)	[28], [36], [37]
	Mozambique		1.39 (0.833–2.00)	0.028 (0–0.083)	$39	4.8 (2.6–9.0)	
	Niger		2.64 (1.39–4.80)	0.053 (0–0.209)	$32	24.0 (11.9–48.2)	

Measles	Benin	98 (95–100)	46.6 (14.5–110)	0.932 (0–4.80)	$73	1.0 (0.3–3.7)	[28], [36], [37]
	Mozambique		181 (56.6–441)	3.62 (0–17.2)	$32	0.4 (0.1–1.3)	
	Niger		26.8 (8.00–65.2)	0.536 (0–2.84)	$25	1.5 (0.4–6.1)	

Pertussis	Benin	80 (70–90)	59.3 (37.3–98.1)	11.9 (6.07–18.1)	$31	0.7 (0.5–1.0)	[28], [36], [37]
	Mozambique		47.2 (30–76.4)	9.44 (4.71–14.6)	$16	0.4 (0.3–0.5)	
	Niger		49.5 (32.4–76.6)	9.90 (4.74–15.7)	$12	1.3 (0.9–1.8)	

Pneumococcal disease	Benin	57 (49–64)	1.05 (0.316–4.42)	0.451 (0.138–1.85)	$111	4.2 (2.1–8.6)	[28], [36], [39], [40]
	Mozambique		1.10 (0.329–4.71)	0.474 (0.148–1.93)	$58	2.4 (1.3–4.3)	
	Niger		0.833 (0.263–3.35)	0.358 (0.105–1.54)	$47	8.7 (4.4–17.5)	

Rotavirus	Benin	50 (35–65)	84.4 (72.3–97.6)	42.2 (25.4–63.3)	$5.53	0.2 (0.1–0.3)	[28], [41], [42], [43], [44]
	Mozambique		91.8 (76.6–109)	45.9 (28.3–67)	$3.33		
	Niger		110 (86.2–140)	54.9 (36.2–75.5)	$2.72		

Rubella CRS	Mozambique	98 (95–100)	0.37 (0.01–0.78)	0	$74	34 (24–39)	[45], [46], [47]
	Niger		1.10 (0–3.60)	0	$58		

TB	Benin	40 (0–80)	0.519 (0.210–2.03)	0.312 (0.181–0.470)	$458	12.3 (6.9–22.2)	[28], [36], [37]
	Mozambique		0.613 (0.310–1.96)	0.368 (0.258–0.470)	$272	6.1 (4.5–8.3)	
	Niger		0.540 (0.230–1.72)	0.324 (0.155–0.510)	$217	24.5 (13.8–43.6)	

Tetanus	Benin	98 (95–100)	0.132 (0.027–0.450)	0.003 (0–0.018)	$316	42.5 (6.7–75.0)	[28], [36], [37]
	Mozambique		0.367 (0.100–0.678)	0.007 (0–0.028)	$161	37.1 (11.0–75.0)	
	Niger		0.565 (0.176–1.48)	0.011 (0–0.060)	$129	42.8 (11.0–75.0)	

Yellow fever	Benin	94 (90–98)	0.247 (0.047–0.853)	0.015 (0.001–0.067)	$10.30	3.4 (0.5–23.0)	[28], [36], [37]
	Niger		0.495 (0.040–2.36)	0.030 (0–0.187)	$4.88	3.3 (0.2–48.9)	

**Table:** 

Pharmaceutical	Usage	Diseases	Dosage^*^	Unit Cost	Refs
Diphtheria antitoxin	Antitoxin	Diphtheria	1 dose of 40–60,000 IU	$15.00 per dose of 10,000 IU (Haffkine Bio)	[38]

Erythromycin	Antibiotic	Diphtheria	14 days at 25 mg/kg 2× daily	$3.47 for 100 tabs of 250 mg (UNICEF)	[48]

Oral rehydration salts	Rehydration	Measles	4–6 days; 80 ml/kg daily	$6.07 for 100 sachets for	[48]
		Yellow fever	5–7 days; 80 ml/kg daily	1 L (UNICEF)	
		Rotavirus	5–7 days; 80 ml/kg daily		
		Hep B	6–10 days; 80 ml/kg daily		

Paracetamol	Fever control	Measles	6–10 days; 15 mg/kg 3–4× daily	$0.83 for 100 tabs of 100 mg (UNICEF)	[48]
		Yellow fever	5–7 days; 15 mg/kg 3–4× daily		
		Pneumococcal	10–12 days; 15/kg 3–4× daily		
		Rubella	6–10 days; 15 mg/kg 3-4x daily		

Amoxicillin	Pneumonia	Measles	5 days; 30 mg/kg 3× daily	$2.24 for 100 tabs of 250 mg (UNICEF)	[48]
		Pneumococcal	10–14 days; 30 mg/kg 3× daily		
		Hib	10–14 days; 30 mg/kg 3× daily		

Tetanus Vaccine	Immune response	Tetanus	Complete series if needed	$1.29 for a 10-dose vial (UNICEF)	[48]

Tetanus Immunoglobulin	Immune response	Tetanus	1 dose of 500 IU	$28.00 per 1500 IU (PAHO EPI)	[31]

Metronidazole	Antitoxin	Tetanus	7 days; 10 mg/kg every 8 h	$0.84 for 100 tabs of 250 mg (UNICEF)	[48]

Diazepam	Anticonvulsant	Tetanus	4–6 days; 0.1–0.3 mg/kg every 1 to 4 h, as needed	$1.50 for 100 tabs of 5 mg (UNICEF)	[48]

Azithromycin	Antibiotic	Pertussis	5 days; 10 mg/kg daily	$0.72 for 5 tabs of 250 mg (UNICEF)	[48]

Ceftriaxone	Antibiotic	Pneumococcal	3–6 days; 50 mg/kg daily	$19.13 for 50 vials of 250 mg (UNICEF)	[48]
		Hib	7 days; 2 g once daily		

Cloxacillin	Antibiotic	Hib (complication)	7 days; 50 mg/kg every 6 h	$9.44 for 500 capsules of 250 mg (UNICEF)	[48]

RHZE fixed dose formulation (TB)	Intensive phase treatment	Tuberculosis	1 dose per 15 kg, daily	$42.99 for 672 tabs (Stop TB)	[32]

RH fixed dose formulation (TB)	Continuation phase treatment	Tuberculosis	1 dose per 15 kg, daily	$20.90 for 672 tabs (Stop TB)	[32]

**Table:** 

Country	Cost per Inpatient Day				Cost per Outpatient Visit			
	Primary	Secondary	% Primary	Weighted Avg	Rural	Urban	% Rural	Weighted Avg
Benin	$16.85	$17.58	80%	$17.00	$3.35	$4.76	53%	$4.01
Mozambique	$8.27	$8.63	80%	$8.34	$2.00	$2.84	65%	$2.29
Niger	$6.48	$6.76	80%	$6.54	$1.67	$2.38	84%	$1.78

**Table:** 

Vaccine-preventable disease	Benin	Mozambique	Niger
Diphtheria	$180.62 ($133.96, $227.27)	$127.83 ($104.93, $150.73)	$116.81 ($98.87, $134.75)
Hep B	$65.68 ($36.29, $95.07)	$32.58 ($18.08, $47.08)	$25.6 ($14.24, $36.96)
Hib	$69.59 ($219.16, $559.11)	$38.36 ($113.53, $280.63)	$31.72 ($91.42, $222.33)
Measles	$60.74 ($34.04, $87.44)	$30.68 ($17.51, $43.85)	$24.34 ($14.02, $34.65)
Pertussis	$30.19 ($16.64, $43.73)	$15.14 ($8.37, $21.91)	$11.87 ($6.57, $17.17)
Pneumococcus	$107.15 ($61.89, $152.42)	$56.1 ($33.81, $78.39)	$45.37 ($27.91, $62.83)
Rotavirus	$5.5 ($3.28, $7.71)	$3.31 ($2.08, $4.54)	$2.71 ($1.75, $3.66)
Rubella	$48.76 ($27.13, $70.39)	$24.29 ($13.67, $34.9)	$19.18 ($10.86, $27.5)
Tuberculosis	$457.68 ($262.66, $652.7)	$272.06 ($136.26, $359.24)	$216.94 ($105.95, $279.31)
Tetanus	$306.21 ($178.49, $433.94)	$156.53 ($93.84, $219.22)	$124.84 ($75.73, $173.96)
Yellow fever	$10.12 ($6.07, $14.17)	$5.85 ($3.72, $7.97)	$4.81 ($3.15, $6.47)

## Appendix C Pharmaceutical unit costs and usage by disease

^*^Reported dosage is relevant for a child of 20 kg except for TB, which is for a 60 kg adult.

## Appendix D Unit costs for care of vaccine preventable disease

Unit costs for a day of inpatient care and for an outpatient visit were calculated based on the WHO-CHOICE regressions [30], updated to 2017 GDP per capita by country. Costs are reported in international dollars.

## Appendix E Direct medical costs per case of vaccine preventable disease

Direct medical costs were calculated for each disease based on the expected hospitalization rate and length of stay, number of expected outpatient visits, and appropriate pharmaceutical usage. Standard clinical protocols were assumed to be consistent across all three countries based on MSF’s clinical guidelines for care in low resource settings. Unit costs for care were country-specific.
